# POLIMI-ITW-S: A large-scale dataset for human activity recognition in the wild

**DOI:** 10.1016/j.dib.2022.108420

**Published:** 2022-06-30

**Authors:** Hao Quan, Yu Hu, Andrea Bonarini

**Affiliations:** a Department of Electronics, Information and Bioengineering, Politecnico di Milano, Piazza Leonardo da Vinci 32, Milano 20133, Italy; b School of Information Engineering, Kaili University, Guizhou Province, China

**Keywords:** Human activity recognition, Computer vision, Mobile robot, In the wild

## Abstract

Human activity recognition is attracting increasing research attention. Many activity recognition datasets have been created to support the development and evaluation of new algorithms. Given the lack of datasets collected in real environments (In The Wild) to support human activity recognition in public spaces, we introduce a large-scale video dataset for activity recognition In The Wild: POLIMI-ITW-S. The fully labeled dataset consists of 22,161 RGB video clips (about 46 h) including 37 activity classes performed by 50 K+ subjects in real shopping malls. We evaluated the state-of-the-art models on this dataset and get relatively low accuracy. We release the dataset including the annotations composed by person tracking bounding boxes, 2-D skeleton, and activity labels for research use at: https://airlab.deib.polimi.it/polimi-itw-s-a-shopping-mall-dataset-in-the-wild.

## Specifications Table

**Table:** 

Subject	Computer Vision and Pattern Recognition
Specific subject area	Human activity recognition
Type of data	2-D RGB video
	Annotation composed by person tracking bounding boxes, 2-D skeleton, and activity labels in JSON format
How the data were acquired	This dataset was taken from RGB cameras of two smartphones with resolution 1920×1080 pixels, 30 fps.
	The models of the two smartphones are *VIVO S7 5G* and *Honor 30 S*.
Data format	Raw
Description of data collection	We collected the dataset in shopping malls in the Hubei province of China. The shopping malls have multiple floors providing different services, e.g., main hall with grocery stores on the ground floor; clothes shops on the second and third floor; restaurants and drink bars on the fourth floor; cinema with a waiting room on the fifth floor; supermarket on the underground floor. The diverse settings guarantee the desired variety of subjects and situations. The subjects are clients and staff in the shopping malls having different genders and ages, including men and women, babies, children, teenagers, adults and elderly people. So we have different subjects for almost every recorded video clip.
	The cameras were held by hands at about 90 cm from the floor. The recorders imitated the mobile robot, keeping moving or staying still by looking around to capture persons that are performing actions.
	We did not mount the cameras on a robot in order to avoid uncommon situations that the presence of a robot may trigger. We did not use 3-D stereo/depth cameras as recording tools since this type of camera suffers for the moving issue, which is not suitable for data collection for mobile robots. Moreover, they may not perform well when subjects are far away from the camera.
Data source location	• Public space: shopping malls
	• City: Shiyan
	• Province: Hubei
	• Country: China
Data accessibility	Repository name: Science Data Bank
	Data identification number: sciencedb.01694
	Direct URL to data: https://doi.org/10.57760/sciencedb.01694

## Value of the Data

• The data are useful for those working in the area of human activity recognition from skeletal data or from RGB videos;
• It will be possible to develop new algorithms to classify a more detailed set of activities based on semantic meanings, thus improving the performance of the applications;
• It could be used to support the development and evaluation of the models of human activity recognition from mobile robots operating in public environments;
• Basing on these data, it will be possible to classify actions performed In The Wild, thus opening a wide sort of applications for Social Robotics and other disciplines;
• Except for the topic of human activity recognition, the dataset could be useful for other human subjects related tasks. Since the clips include many crowded scenes, this dataset is interesting for investigating opening problems like person tracking, pose tracking, person re-identification, body/head orientation.
• The data may also contribute to the autonomous robotic research field to develop new path planning, and obstacle avoidance methods.
• Other researchers will become interested in problems and algorithms arising when operating in the wild.

## 1 Data Description

Human activity recognition (HAR) involves skeleton representations of human bodies instead of raw RGB videos. Due to its strong adaptability and highly abstract characteristics, many significant models were developed based on skeletal data [1], [2], [3], [4], [5], [6], [7]. Compared to the RGB video representation, the greatest benefits of the skeletal data are that they are free of dynamic environment noise and robust against complicated backgrounds (lighting conditions, color of clothing, object obstruction, etc.). It is important for service robots to recognize the actions of people in the real world to further enhance their capabilities to offer services.

We analyzed some relevant skeleton-based HAR models in the last three years to check how public datasets were used to train and evaluate models in the community. As shown in Table 1, the most commonly used datasets are (in descending order): NTU RGB + D 60 [8], NTU RGB + D 120 [9], Kinetics [10], Northwestern-UCLA Multiview Action 3D [11], SYSU 3D Human-Object Interaction [12] datasets. Among those, only Kinetics was not collected from a constrained environment but from online streaming resources by using the crowd-sourcing method instead, while all the other datasets were collected in the respective laboratories.

**Table 1:** Datasets used for recent human recognition models.

Model	Publisher	NTU 60 [8]	NTU 120 [9]	Kinetics-Skeleton [10], [13]	NUCLA [11]	SYSU [12]
Efficient GCN [14]	TPAMI 22	✓	✓			
CTR-GCN [7]	ICCV 21	✓	✓		✓	
SGN [5]	CVPR 20	✓	✓			✓
MSG3D [3]	CVPR 20	✓	✓	✓		
4S-Shift-GCN [15]	CVPR 20	✓	✓		✓	
NAS-GCN [4]	AAAI 20	✓		✓		
2S-AGCN [1]	CVPR 19	✓		✓		

**Total**		7	5	3	2	1

The state-of-the-art models got about 90% accuracy on the datasets collected in laboratory environments as shown in Table 2, 3, 5 and 6. Nevertheless, they got only less than 40% accuracy on the Kinetics dataset which was collected from online streaming resources by crowd-sourcing methods as shown in Table 4. It hints that the state-of-the-art models could perform well on the datasets collected in constrained environments, but they may meet challenges when recognizing actions from unconstrained, natural environments.

**Table 2:** The state-of-the-art methods on NTU 60 dataset in accuracy (%).

NTU 60: collected from laboratory			
Model	Publisher	X-Sub^a^ (%)	X-View^b^ (%)
Efficient GCN [14]	TPAMI 22	92.1	96.1
CTR-GCN [7]	ICCV 21	92.4	96.8
MS-G3D [3]	CVPR 20	91.5	96.2
4S-Shift-GCN [15]	CVPR 20	90.7	96.5
SGN [5]	CVPR 20	89.0	94.5
NAS-GCN [4]	AAAI 20	89.4	95.7
2S-AGCN [1]	CVPR 19	88.5	95.1

**Average Value**		90.5	95.8

a X-Sub: Cross-Subject evaluation [8].

b X-View: Cross-View evaluation [8].

**Table 3:** The state-of-the-art methods on NTU 120 dataset in accuracy (%).

NTU 120: collected from laboratory			
Model	Publisher	X-Sub120^a^ (%)	X-Set120^b^ (%)
Efficient GCN [14]	TPAMI 22	88.7	88.9
CTR-GCN [7]	ICCV 21	88.9	90.6
MS-G3D [3]	CVPR 20	86.9	88.4
4S-Shift-GCN [15]	CVPR 20	85.9	87.6
SGN [5]	CVPR 20	79.2	81.5
**Average Value**		85.9	87.4

a X-Sub120: Cross-Subject evaluation [9].

b X-Set120: Cross-Setup evaluation [9].

**Table 4:** The state-of-the-art methods on Kinetics-Skeleton dataset in accuracy (%).

Kinetics-Skeleton: collected by crowd-sourcing method		
Model	Publisher	Kinetics-Skeleton (%)
MS-G3D [3]	ICCV 21	38
NAS [4]	CVPR 20	37.1
2S-AGCN [1]	CVPR 19	36.1
**Average Value**		37.1

**Table 5:** The state-of-the-art methods on NUCLA dataset in accuracy (%) .

NUCLA: collected from laboratory		
Model	Publisher	NUCLA (%)
CTR-GCN [7]	ICCV 21	96.5
4S-Shift-GCN [15]	CVPR 20	94.6
**Average Value**		95.6

**Table 6:** The state-of-the-art method on SYSU dataset in accuracy (%).

SYSU: collected from laboratory			
Model	Publisher	X-Sub^a^ (%)	Same-Sub^b^ (%)
SGN [5]	CVPR 20	90.6	89.3

a X-Sub: Cross-Subject evaluation [12].

b Same-Sub: Same-Subject evaluation [12].

Because the main datasets for evaluating new HAR models are collected in the specific laboratories and the accuracy is about 90%, we think that there is little optimizing space for the models trained on such a type of datasets. Meanwhile, we argue that reliable HAR models to support the production of mobile service robots should not only be evaluated on the datasets collected in controlled environments but also on datasets collected in the final, public environments, situation defined in the community as “In The Wild” (ITW). Due to the well-known issues (like having unbalanced taxonomies, unnatural scenes, label noise and invalid websites links) of the crowd-sourcing methods, the dataset like Kinetics collected from online streaming resources by crowd-sourcing methods may not satisfy the needs of developing robust models which are able to perform well in the real world.

To fill this gap, we propose the POLIMI-ITW-S dataset to develop reliable skeleton-based human activity recognition models that could be deployed on mobile service robots to recognize actions that happen in the real world.

We propose that a reliable ITW dataset of clips useful for robotic applications should have the following characteristics:

1. viewpoint similar to the one of the robot, including subjects viewed both in full figure when the robot is far from the subject and only in part, when the robot is close, taken from a camera having characteristics typical of the ones that are mounted on commercial, mobile, service robots;
2. video clips recorded from free moving viewpoints like the ones of mobile robots;
3. clips representative of the common actions in the selected environment, evenly distributed among the classes;
4. a large number of different subjects performing the same action;
5. different genders of subjects with a large range of ages, from babies to elderly people;
6. real-life background, possibly including people and objects that could typically be in the context;
7. presence of crowded scenes with large quantities of persons, including subjects occluded by person(s) or object(s).
8. unscripted, natural actions: there are no “actors”, people are recorded without knowing in advance that they are, so they are supposed to perform naturally;
9. real-life sequencing of actions, so that it is possible to consider typical sequences of actions from realistic clips;
10. possible inclusion of human-object and multi-person interactive actions (such as *“calling”, “talking”, “drinking”, “eating”, “holding baby in arms”*, etc.);
11. large-scale dataset.

As shown in Table 7, the available datasets are usually collected in controlled contexts, such as laboratory, home, or conveniently extracted from streaming sources produced for other purposes.

**Table 7:** Comparison between different datasets and *ITW* dataset requirements: 1. viewpoint similar to the one of the robot, 2. video taken from moving camera, 3. representative actions, 4. different people performing the same action, 5. different genders and ages, 6. real life background, 7. crowded scenes with occlusions, 8. no “actors” and unscripted actions, 9. presence of sequences of actions, 10. presence of human-object and multi-agent interactive actions, 11. large-scale dataset.

Datasets	Year	Classes	Subjects	Samples	Scenes	Views	1	2	3	4	5	6	7	8	9	10	11
SYSU [12]	2015	12	40	480	1	1	Y	N	Y	Y	N	N	N	N	N	N	N
ActivityNet [16]	2015	203	–	849 h	–	1	N	Y	N	Y	Y	Y	Y	Y	Y	Y	Y
NTU [8]	2016	60	40	56,880	1	3	Y	N	N	Y	N	N	N	N	N	Y	Y
Kinetics [10]	2017	400	–	300,000	–	1	N	Y	N	Y	Y	Y	Y	Y	Y	Y	Y
AVA [17]	2018	80	–	437	–	1	N	Y	N	Y	Y	Y	Y	N	Y	Y	Y
NTU 120 [9]	2019	120	106	114,480	1	3	Y	N	N	Y	N	N	N	N	N	Y	Y
Toyota S.H. [18]	2019	31	18	16,115	1	7	N	N	Y	Y	N	Y	N	Y	Y	N	Y
ETRI [19]	2020	55	100	112,620	1	4	Y	N	Y	Y	N	Y	N	Y	Y	Y	Y
FineGym [20]	2020	530	–	708 h	–	1	N	Y	N	Y	N	Y	N	Y	Y	Y	Y
BABEL [21]	2021	256	–	43.5 h	1	1	Y	N	Y	N	N	N	N	N	Y	Y	Y
UAV-Human [22]	2021	155	Multiple	67,428	–	1	N	Y	Y	Y	Y	Y	N	Y	Y	Y	Y
HOMAGE [23]	2021	75	27	25.4 h	–	2–5	N	N	Y	Y	Y	Y	N	N	Y	Y	Y
POLIMI-ITW-S	2022	37	50 K+	233,446 ∼46 h	malls	robotic	Y	Y	Y	Y	Y	Y	Y	Y	Y	Y	Y

The main advantages of these datasets are that they are easy to obtain and with relatively low labor cost comparing to the datasets collected in public spaces. In addition, datasets creators could take advantages of the limited environments to deploy RGB-D cameras to get 3-D human skeletal data. Since the actors know what they should do in advance and there are rarely considered crowded scenes, they did not need to dedicate a lot of resources for annotation.

However, most datasets do not consider the viewpoint of mobile robots in public spaces. Furthermore, there is no large-scale visual dataset that deals with real, daily behavior of people. In most cases, actions are performed upon request, often by actors, usually separated from each other. Results obtained starting from such constrained conditions may not completely hold in a real-world scenario, as we have verified on our dataset for state of art models. There is a lack of adequate dataset to train models that could be used by robots to recognize common human activities in public spaces. The absence of datasets for human activity recognition in the wild is a serious impediment to computer vision and robot intelligence research.

Different from the state of art datasets, our dataset satisfies all the requirements mentioned above for a reliable ITW dataset.

We have collected 22,161 video clips with more than 15.4 million frames. The average duration of each video clip is about 7 s. The total length of the dataset is about 45.97 h. The dataset was collected in Hubei province of China. According to the population statistics published by the local government [24], the gender distribution of the city is 51.48% (male) and 48.52% (female). The age distribution is 18.7% (0–14), 62.28% (15–59), 19.03% (over 60). We believe the distribution of gender and age in the dataset matches the distribution of the population of the city. Individuals were anonymized by blurring faces using RetinaFace [25]. We used OpenPifPaf [26] to extract person tracking bounding boxes and 2-D skeleton data.

Before starting the annotation work, we analyzed a subset of the collected video clips and used the proposed detailed labeling mode to define 37 activity classes. Actually, except for the defined activity classes, there are also other activities that occurred in videos such as “falling down”, “fighting”, “kicking”, “throwing trash”, etc. We didn’t add these activities to the dataset since they have a relatively small number of clips, which would have dramatically affected the learning performance.

The defined classes were distributed on three levels: The labels of the *general* level are used for describing single actions. We have defined *“standing”, “walking”, “sitting”, “crouching”, “cleaning”, “jumping”, “laying”, “riding”, “running” and “scooter”* for this level. The *modifier* level labels are *“walkingTogether”, “sittingTogether”, “standingTogether”, etc*, which refer to multiple persons or a group of people walking, sitting, or standing together, etc. The *aggregate* level detailed labels aim at describing multiple actions in a single label, such as *“standingWhileCalling”, “standingWhileLookingAtShop”, “walkingWhileWatchingPhone”, “sittingWhileHoldingBabyInArms”*, etc. The complete list of the defined labels is shown in Table 8.

**Table 8:** Activity labels.

**General Level (10)**:
cleaning, crouching, jumping, laying, riding, running, scooter, sitting, standing, walking
**Modifier Level (3)**:
sittingTogether, standingTogether, walkingTogether
**Aggregate Level (24)**:
sittingWhileCalling, sittingWhileDrinking, sittingWhileEating, sittingWhileHoldingBabyInArms,
sittingWhileTalkingTogether, sittingWhileWatchingPhone, standingWhileCalling, standingWhileDrinking,
standingWhileEating, standingWhileHoldingBabyInArms, standingWhileHoldingCart, standingWhileHoldingStroller,
standingWhileLookingAtShops, standingWhileTalkingTogether, standingWhileWatchingPhone, walkingWhileCalling,
walkingWhileDrinking, walkingWhileEating, walkingWhileHoldingBabyInArms,
walkingWhileHoldingCart, walkingWhileHoldingStroller, walkingWhileLookingAtShops,
walkingWhileTalkingTogether, walkingWhileWatchingPhone

We have also defined a rule for the labels containing the keyword “together”. It is only used for the groups of persons performing social activities. For example, if two or more persons are standing closely, but are not involved in any social activities, their activities will not be considered as done “together”.

The dataset was fully labeled by HAVPTAT [27]. We provide RGB videos, persons’ tracking bounding boxes, 2-D skeleton data (17 body keyjoints) and labeled activities’ classes in JSON format. To build a high-quality dataset that offers correct annotations, we adopt a series of approaches including: training annotators with tutorial slides and video demos, pre-testing the annotators rigorously before formal annotation, and cross-validating across annotators.

For the reader’s convenience, Table 9 shows the COCO body 17 keypoint arrangement [28], [29] adopted in our dataset. We notice that the entire dataset misses joints 4 (left ear) and 5 (right ear) of the COCO’s 17 keypoints.

**Table 9:** COCO body keypoints .

1	Nose
2	left eye
3	right eye
4	left ear
5	right ear
6	left shoulder
7	right shoulder
8	left elbow
9	right elbow
10	left wrist
11	right wrist
12	left hip
13	right hip
14	left knee
15	right knee
16	left ankle
17	right ankle

**Fig. 1:**
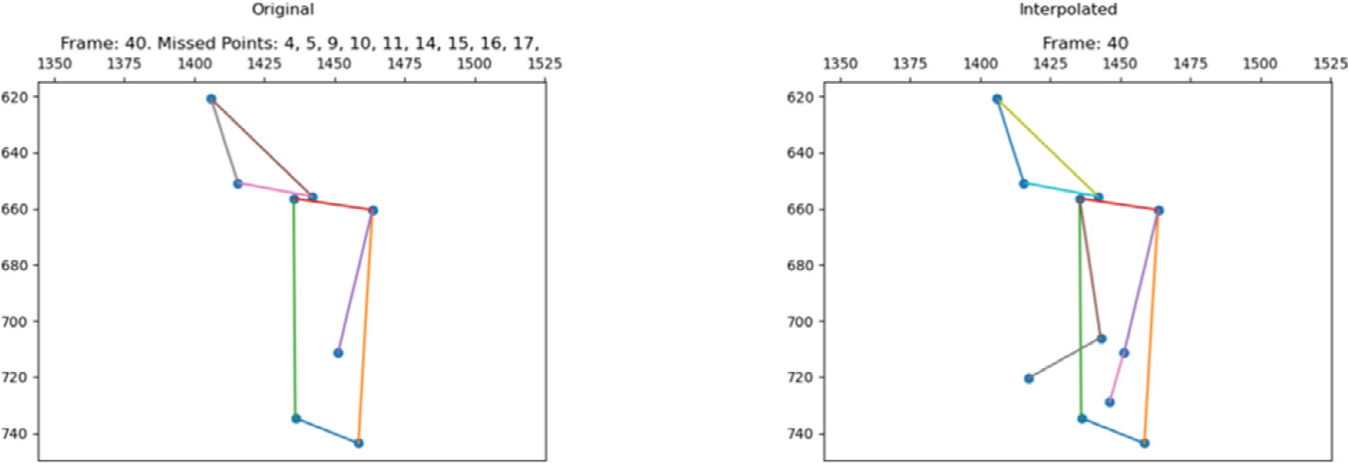
Original pose (left); Pose reconstructed by “interpolation” (right).

The skeletons of the data collected from the real world are often incomplete. A frame containing few joints could lead the ambiguity for the learning model. For instance, a frame with only 2 and 3 joints may reduce the possibility for a system to identify the corresponding activity class. To reduce such a type of learning error, we also temporal-linearly interpolated the missing joints and hold the pose as valid only with more than a given number (nine) joints. The threshold was fixed by nine since only partial bodies could be captured by a camera in some cases. For example, as shown in Fig. 1, when the camera is close to a person, only the upper part of the body is present, but the keypoints of the lower part of the body (14 left knee, 15 right knee, 16 left ankle, and 17 right ankle) miss. The left picture of Fig. 1 is the original pose extracted by OpenPifPaf [26]. Right elbow (9), left (10) and right (11) wrist were not detected. After having been processed by the interpolation operation provided by the Python’s Pandas library [30], the three missing keypoints were reconstructed on the right picture. This approach was applied to the entire dataset. We call it the “original version” dataset in the experimental phase.

**Fig. 2:**
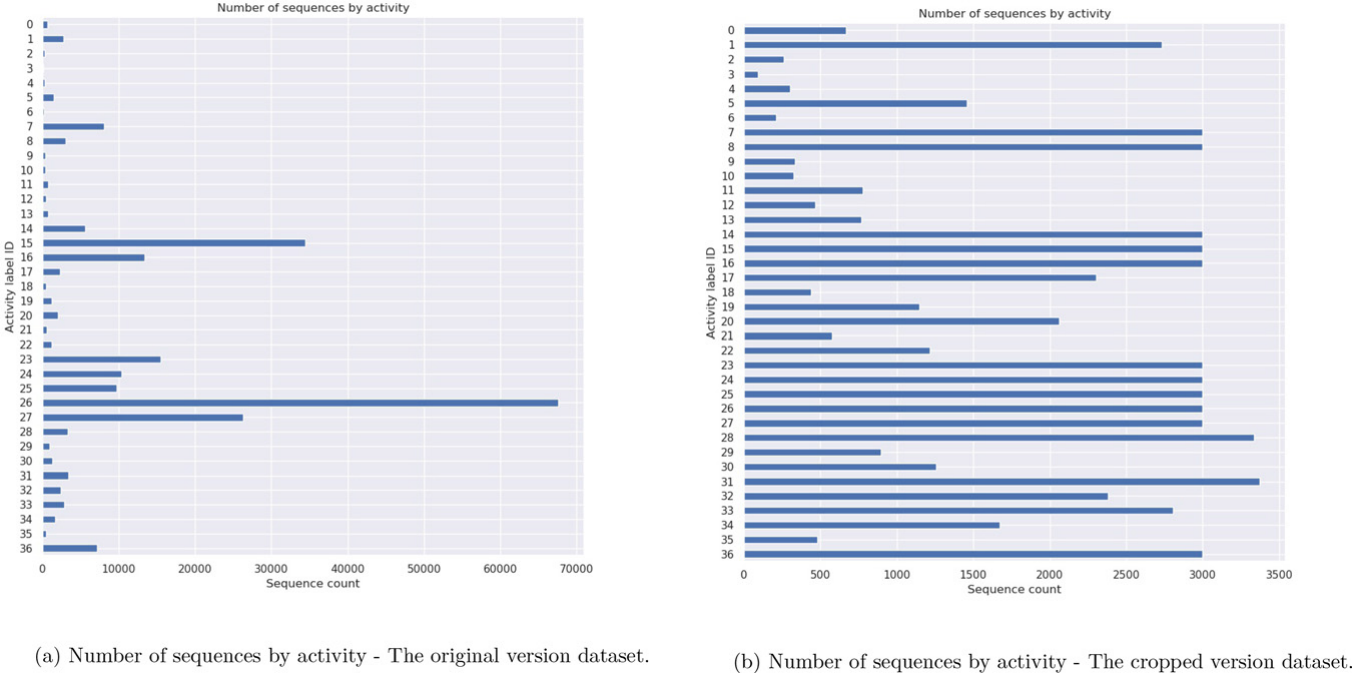
Number of sequences by activity.

From Table 10 and Fig. 2a, we could see that the imbalance issue is present in the original dataset. The most frequent activities are *“walking”, “standing”*, and *“walkingTogether”*, which occur in about 55% of the dataset.

**Table 10:** Number of sequences and frames in the original and the cropped versions of datasets.

Label ID	Activity class	#Seq. orig.	#Frame orig.	#Seq. crop.	#Frame crop.
0	cleaning	665	60,814	665	60,814
1	crouching	2735	195,537	2735	195,537
2	jumping	260	12,564	260	12,564
3	laying	92	7071	92	7071
4	riding	301	13,555	301	13,555
5	running	1457	63,498	1457	63,498
6	scooter	208	11,919	208	11,919
7	sitting	8070	376,905	3000	182,026
8	sittingTogether	3044	195,566	3000	193,984
9	sittingWhileCalling	334	42,693	334	42,693
10	sittingWhileDrinking	325	32,778	325	32,778
11	sittingWhileEating	776	81,780	776	81,780
12	sittingWhileHoldingBabyInArms	467	34,998	467	34,998
13	sittingWhileTalkingTogether	766	82,371	766	82,371
14	sittingWhileWatchingPhone	5602	546,327	3000	357,020
15	standing	34,399	1,785,446	3000	158,669
16	standingTogether	13,367	902,424	3000	202,879
17	standingWhileCalling	2303	307,560	2303	307,560
18	standingWhileDrinking	439	46,009	439	46,009
19	standingWhileEating	1148	125,342	1148	125,342
20	standingWhileHoldingBabyInArms	2059	144,727	2059	144,727
21	standingWhileHoldingCart	576	44,719	576	44,719
22	standingWhileHoldingStroller	1216	121,875	1216	121,875
23	standingWhileLookingAtShops	15,524	1,193,938	3000	220057
24	standingWhileTalkingTogether	10,310	1,032,687	3000	362779
25	standingWhileWatchingPhone	9727	990,380	3000	268797
26	walking	67,615	3,384,638	3000	142640
27	walkingTogether	26,276	1,621,113	3000	179645
28	walkingWhileCalling	3338	401,582	3338	401,582
29	walkingWhileDrinking	896	86,520	896	86,520
30	walkingWhileEating	1256	128,535	1256	128,535
31	walkingWhileHoldingBabyInArms	3373	198,582	3373	198,582
32	walkingWhileHoldingCart	2381	206,911	2381	206,911
33	walkingWhileHoldingStroller	2806	268,606	2806	268,606
34	walkingWhileLookingAtShops	1674	94,657	1674	94,657
35	walkingWhileTalkingTogether	479	38,259	479	38,259
36	walkingWhileWatchingPhone	7182	581,222	3000	195,973
Total		233,446	15,464,108	65,330	5317931

**Fig. 3:**
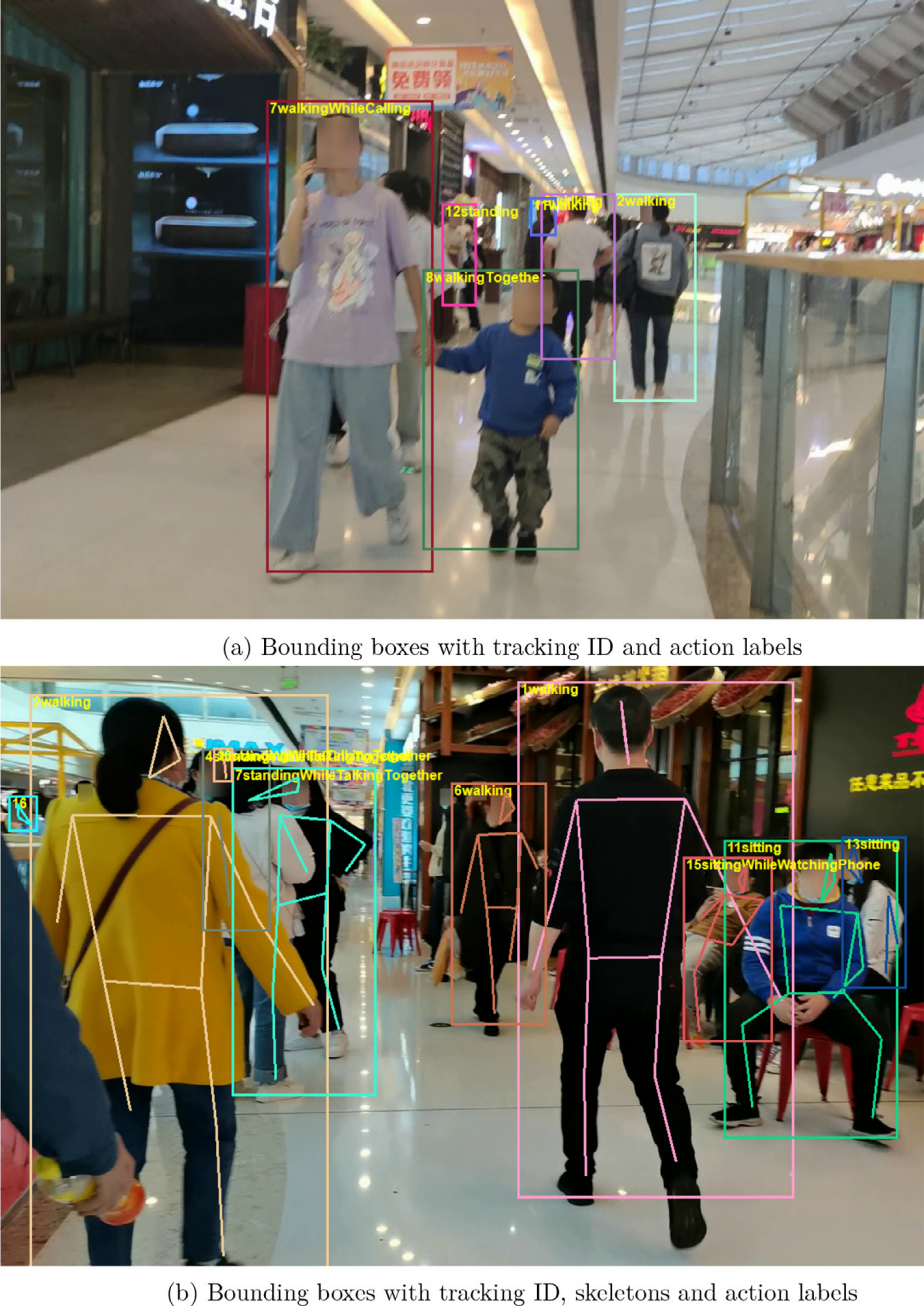
Annotation samples.

We show two snapshots with annotation in Fig. 2. Fig. 3a shows bounding boxes with tracking ID and annotated action labels. Fig. 3b shows the same information and skeletons.

## 2 Experimental Design, Materials and Methods

**Fig. 4:**
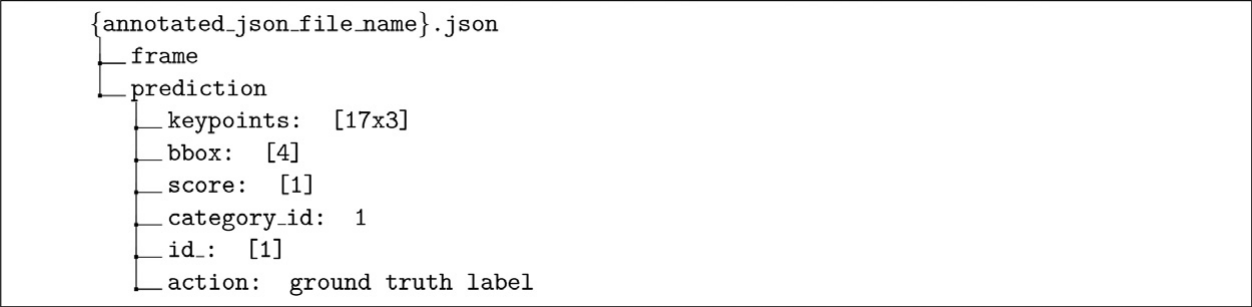
The structure of annotated JSON format file.

**Fig. 5:**
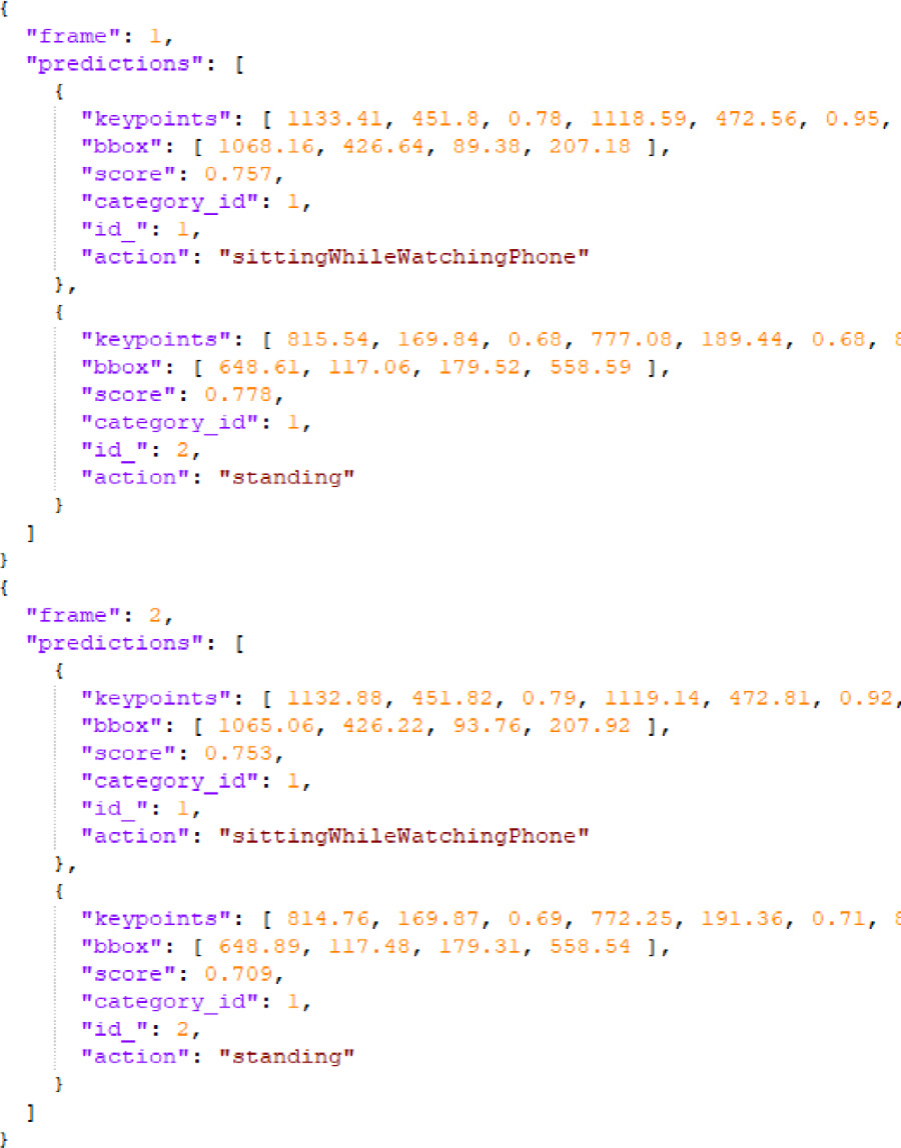
An annotation example.

The structure of the annotated JSON format file and the data information of the fields are shown in Fig. 4. An annotated file is composed by all the information of frames ordered by temporal sequence. Every “frame” contains the main entry “prediction” which includes the detailed data: “keypoints” are composed by 17 tuples of (*X, Y*, confident_score) with *X, Y* coordinates and a confident score of each joint (in total [17×3] dimensional data shape); “bbox” is composed by upper left *X, Y* coordinates, width, height of the bounding box ([4] dimensional data shape), “score” is the confident score of the bounding box ([1] dimensional data shape), “category_id” is the constant 1 inferring a person subject following the convention of COCO annotation ([1] dimensional data shape), “id_” is the ID of a tracked person ([1] dimensional data shape), “action” is the ground truth label. A piece of an annotation file is shown in Fig. 5. The example is composed by two frames of a clip and each frame includes two persons with “sittingWhileWatchingPhone” and “standing” actions.

We used PyHAPT to pre-process the data [31].^1^, ^2^ After the annotation work done by HAVPTAT [27], we obtained the annotated files like the example shown in Fig. 5. We thus represent each joint with a couple of pairs (*X, Y*) corresponding to its extremes so that a skeleton frame is recorded as an array of 17 couples with data shape (17, 2). Based on the field of “id_” which is the person tracking ID, we could facilitate composing the keypoints of the same person in different *T* temporal frames to get (*T*, 17, 2) data shape. For the multi-person cases, we take all the detected persons in each clip into account. We consider each person performing the same action in a single video clip as a valid action sequence. If the same person performs multiple actions in a single video clip, we consider them as different action sequences performed by the same person. Since every action sequence includes only a person’s data, so we extend the previous data shape to (*T*, 17, 2, 1) for convenience of implementation. For the whole dataset, the script reshapes and gets the array of (*N*, 2, *T*, 17, 1) dimensions by concatenating the single action sequences of persons with *N* action samples. We summarize the meaning of each element in the tuple: the script generates *N* samples of action sequences in total; two dimensions (*X, Y*) skeletal data; an action sequence lasts *T* frames; 17 keypoints of a human body; 1 person data in each tuple. All action skeleton sequences are padded to T=300 frames by replaying the actions as also done by other skeletal datasets. The training set and test set split ratio is 70% and 30%. The number of padded frames and the training-test set split ratio can be both customized by users.

From Table 1, we evaluated four most relevant state-of-the-art human activity recognition models in the last three years (Efficient GCN [14], CTR-GCN [7], MS-G3D [1], 2S-AGCN [3]) on the new POLIMI-ITW-S dataset. We believe the results are representative for the current mainstream human activity recognition algorithms.

To have a fair comparison, we decided to use joint data for training and test. As a result, from Table 11, we observe that the accuracy is only less than 50%. We infer that the state-of-the-art activity recognition models could not perform well on real-life data.

**Table 11:** The results of skeleton-based activity recognition.

Model	Publisher	Accuracy (%)	
		Original	Cropped
Efficient GCN [14]	TPAMI 22	48.3	38.5
CTR-GCN [7]	ICCV 21	44.93	34.97
MS-G3D [3]	CVPR 20	43.37	34.05
2S-AGCN [1]	CVPR 19	44.46	34.13

When we were in the data collection phase, we already noticed that most of the actions occurred are *“walking”* and *“standing”* without involving any other additional actions in the shopping malls. This leads to an imbalance issue: some highly frequent actions appear more often than others. For instance, *“walking”* and *“standing”* have 80 K+ and 50 K+ sequences, while *“walkingWhileEating”* and *“sittingWhileDrinking”* have only 1256 and 325 sequences.

To evaluate whether the imbalance classes could cause the bad performance, we tried to take only 3000 sequences of some classes with huge numbers to build a “cropped” version dataset as shown in Fig. 2b. We evaluated the models also on the “cropped” version dataset. Unfortunately, the accuracy was even lower than the one with the original version dataset. We could say that the imbalanced classes might not directly affect the performance of the models.

## Ethics Statements

According to the GDPR Art. 89, individuals were properly de-identified by blurring their faces. The dataset can be used for research purposes.

## CRediT authorship contribution statement

**Hao Quan:** Data curation, Software, Writing – original draft, Investigation, Formal analysis. **Yu Hu:** Data curation, Investigation. **Andrea Bonarini:** Conceptualization, Methodology, Validation, Writing – review & editing, Supervision.

## Declaration of Competing Interest

The authors declare that they have no known competing financial interests or personal relationships that could have appeared to influence the work reported in this paper.

## Data Availability

POLIMI-ITW-S: A shopping mall dataset In-The-Wild (Original data) (Mendeley Data).

## Footnotes

1 https://github.com/AIRLab-POLIMI/PyHAPT

2 CodeOcean reproducible capsule: https://doi.org/10.24433/CO.6886084.v1
